# Single cell‐type transcriptome profiling reveals genes that promote nitrogen fixation in the infected and uninfected cells of legume nodules

**DOI:** 10.1111/pbi.13778

**Published:** 2022-01-31

**Authors:** Longlong Wang, Yu Zhou, Runhui Li, Jianjun Liang, Tao Tian, Jie Ji, Runzhou Chen, Yumiao Zhou, Qiuling Fan, Guogui Ning, Robert M. Larkin, Manuel Becana, Deqiang Duanmu

**Affiliations:** ^1^ State Key Laboratory of Agricultural Microbiology Hubei Hongshan Laboratory Huazhong Agricultural University Wuhan China; ^2^ Key Laboratory of Horticultural Plant Biology Ministry of Education Huazhong Agricultural University Wuhan China; ^3^ Departamento de Nutrición Vegetal Estación Experimental de Aula Dei Consejo Superior de Investigaciones Científicas Zaragoza Spain

**Keywords:** haem, leghaemoglobin, legume nodules, single‐cell transcriptomics, symbiotic nitrogen fixation

Excessive application of nitrogen fertilizers has inevitably resulted in environmental problems. The symbiotic nitrogen fixation (SNF) that occurs in the root nodules of leguminous plants provides a sustainable source of reduced nitrogen in agricultural ecosystems. More than 200 genes have been reported to regulate SNF, including rhizobial infection, nodule organogenesis and senescence (Roy *et al*., 2020). Mature nodules consist mainly of two cell types: infected cells (IC) that contain nitrogen‐fixing bacteroids and uninfected cells (UC) that mediate active metabolism and nutrient transport. Although it is well known that SNF requires functional specialization, the specific genes responsible for transcriptional regulation and carbon/nitrogen metabolism and transport in IC and UC remain largely unexplored.

Single‐cell transcriptomics has emerged as a powerful technique for investigating spatiotemporal patterns of gene expression. The maximum cell diameter acceptable for these droplet‐based methods is ~40 μm. An average size of IC from a legume nodule is typically 50–100 μm. We therefore manually separated reddish‐brown IC and transparent UC (50–100 cells for each of these two cell types) from the mature nodules of *Lotus japonicus* at 4 weeks post‐inoculation (wpi) with *Mesorhizobium loti* MAFF 303099 (Figure 1a,b; Appendix S1). Due to insurmountable technical difficulties, we were unable to separate IC into cells having various levels of nuclear DNA endoreduplication, or UC into outer and inner cortical cells, vascular bundle cells and interstitial cells, typically found in determinate nodules. Smart‐Seq2 libraries were constructed (Picelli *et al*., 2014), and samples with >50% mapping rates were used for the transcriptome analysis, including four replicates for UC and two replicates for IC (Figure 1c). We finally obtained 939 DEGs (|Log_2_‐fold change (IC vs. UC)| > 3, FDR < 0.05; Table S1). Of the detected genes, 925 had not been previously characterized, and therefore, our transcriptomic analysis provides a resource to study SNF in mature nodules (Roy *et al*., 2020). Notably, we found 55 genes that encode putative transcription factors and 73 genes that encode transporters (Figure 1d).

**Figure 1:**
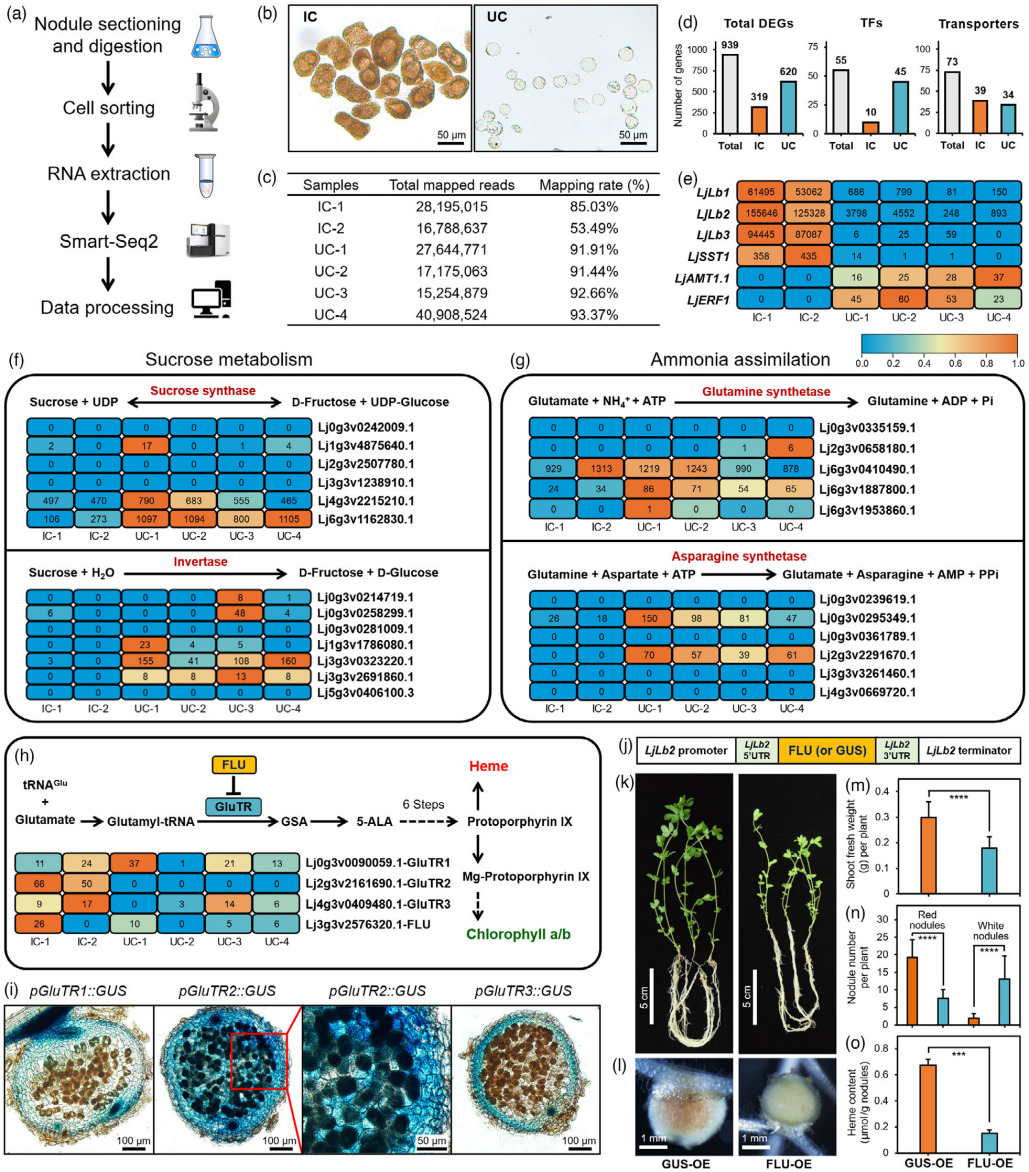
Genetic characterization of cell‐type‐specific components in root nodules. (a) Flowchart of sample preparation and single cell‐type transcriptome analysis in nodules from *L. japonicus* at four weeks post‐inoculation (wpi) with rhizobia. (b) Morphological comparison of infected cells (IC) and uninfected cells (UC). (c) Numbers of mapped reads and the mapping rate in six samples. (d) Number of differentially expressed genes (DEGs). (e) Expression patterns of selected SNF‐related genes. The number represents fragments per kilobase of transcript per million mapped reads (FPKM). (f–h) Expression patterns of genes involved in sucrose metabolism (f), ammonia assimilation (g) and haem biosynthesis (h). Heatmaps in (e–h) were illustrated using the zero‐to‐one method at each row scale using TBtools, setting the maximal FPKM value of each gene to one. (i) Expression of the *pGluTR::GUS* in nodules at 3 wpi. (j) Illustration of constructs for the overexpression of FLU or GUS. (k–o) Symbiotic phenotypes of plants expressing FLU (29 plants) or GUS (23 plants) at 5 wpi, including growth phenotype (k), nodule morphology (l), shoot fresh weight (m), number of red and white nodules (n) and haem content (o). Bars indicate mean values ± SD. ^***^ (*P* < 0.001) and ^****^ (*P* < 0.0001) indicate significant differences based on Student’s *t*‐test. FLU, fluorescence in blue light; GluTR, glutamyl‐tRNA reductase; 5‐ALA, 5‐aminolevulinic acid; GSA, glutamate‐1‐semialdehyde.

We found that several genes previously linked to SNF, such as the leghaemoglobin genes (*LjLbs*) and a sulphate transporter gene (*LjSST1*), are expressed at high levels in IC. Interestingly, ~2% of the total *Lb2* mRNA was detected in UC, which is consistent with a recent study showing promoter activity of that gene in the interstitial cells of *L. japonicus* nodules (Wang *et al*., 2019). In contrast, we found that an ammonia transporter (*LjAMT1.1*) and an ERF transcription factor (*LjERF1*) are exclusively expressed in UC (Figure 1e; Roy *et al*., 2020). Next, we determined the expression profiles of genes involved in carbon and nitrogen metabolism. Sucrose synthase and invertase are key enzymes involved in sucrose metabolism. We found that two of the six sucrose synthase genes (Lj4g3v2215210.1 and Lj6g3v1162830.1) and one of the seven invertase genes (Lj3g3v0323220.1) are expressed at higher levels in nodules, predominantly in UC relative to IC (Figure 1f). These results support the proposal that sucrose is transported to UC and catabolized there to dicarboxylates (White *et al*., 2007).

The nitrogenase enzymatic complex reduces N_2_ to ammonia in IC. Glutamine synthetase (GS) and asparagine synthetase (AS) are two key enzymes for the assimilation of ammonia into glutamine and asparagine, which serve as the two major forms of fixed nitrogen being translocated to the shoot in *L. japonicus* (Miao *et al*., 1991). Two of the five genes encoding GS (Lj6g3v0410490.1 and Lj6g3v1887800.1) were found to be highly expressed in nodules in both UC and IC (Figure 1g). Our finding that the expression of an ammonia transporter (*LjAMT1.1*) is enhanced in UC supports the notion that UC may contribute to ammonia assimilation (Figure 1e; Rogato *et al*., 2008). Additionally, two of the six *AS* genes encode the main AS isoforms in nodules and were found to be highly expressed in UC relative to IC (Figure 1g). These results show that asparagine is mainly synthesized in UC. Unexpectedly, we found that glutamine might be actively synthesized in both IC and UC. The significance of glutamine biosynthesis in UC is currently unknown.

Haem is the prosthetic group of Lbs and is synthesized by the tetrapyrrole pathway. Glutamyl‐tRNA reductase (GluTR) catalyses the rate‐limiting step in haem biosynthesis and is controlled by complex mechanisms at the transcriptional and post‐translational levels (Figure 1h; Czarnecki and Grimm, 2012). The *L. japonicus* genome harbours three *GluTR* genes. We found that *LjGluTR2* is exclusively expressed in IC. In contrast, no apparent expression patterns were observed for *LjGluTR1* and *LjGluTR3* (Figure 1h). We then performed a promoter‐GUS fusion analysis and found that only the expression of *LjGluTR2* is enhanced in IC (Figure 1i). Thus, our results strongly support that LjGluTR2 is the main isoform responsible for haem biosynthesis in nodules.

Previous studies have shown that Arabidopsis FLU (fluorescence in blue light) regulates haem biosynthesis by inhibiting GluTR activity (Hou *et al*., 2019; Figure 1h). To investigate the biological function of FLU in nodules, we used the IC‐enhanced *LjLb2* promoter (~3 kb) to overexpress the *FLU* gene (Wang *et al*., 2019; Figure 1j). Plants overexpressing *FLU* developed white nodules and yellow leaves, in contrast to the GUS‐expressing control plants (Figure 1k,l). The *FLU* overexpressing plants exhibited lower shoot fresh weight, produced fewer red nodules and more white nodules, and had lower haem content in the nodules (Figure 1m–o). These data indicate that the post‐translational regulation of haem biosynthesis is essential for SNF.

In summary, this study identified specific genes that contribute to the distinct activities of IC and UC in N_2_‐fixing nodules. The majority of these genes have not been previously characterized (Roy *et al*., 2020), laying the foundation for future investigations on the contributions of these genes to SNF by influencing transcription, metabolism and metabolite transport inside the nodules.

## Conflicts of interest

The authors declare no conflicts of interest.

## Author contributions

DD and YZ designed the research. LW, YZ, JL, JJ, TT, Y‐MZ and QF performed the experiments. RL, RC and GN performed the bioinformatics analysis. LW, RML, MB and DD wrote and revised the manuscript.

## Supporting information


**Appendix S1** Protocol for isolating infected and uninfected cells from *Lotus japonicus* nodules.

**Table S1** ​Transcripts abundance of *L. japonicus* genes in IC and UC.

## Data Availability

Transcriptomic data were deposited in NCBI under accession number GSE188748.
