# Supplementary material for: Single cell‐type transcriptome profiling reveals genes that promote nitrogen fixation in the infected and uninfected cells of legume nodules
Source: Plant Biotechnol J. 2022 Jan 31;20(4):616–8. doi: 10.1111/pbi.13778 (PMC8989494; doi:10.1111/pbi.13778)
Supplement: Supplementary file 1 — Appendix S1 Protocol for isolating infected and uninfected cells from Lotus japonicus nodules. [file PBI-20-616-s001.pdf]

Protocol for isolating infected and uninfected cells from *Lotus japonicus* nodules

Part A: Preparation of nodule protoplasts

1. Mature root nodules (~1 mm diameter) were collected from wild-type MG-20 plants of *Lotus japonicus* at 4 wpi (Figure S1A, B).
2. Approximately 1 g of nodules were hand-sliced with ~0.5 mm thickness using a razor blade on a sterile disposable petri dish.
3. Nodule slices were vacuum-infiltrated for 15 min in enzyme solution (1.5% cellulose R-10, 0.4% macerozyme R-10, 450 mM mannitol, 20 mM MES (pH 5.7), 10 mM $CaCl_2$, 20 mM KCl, 0.1% BSA, 10 mM ribonucleoside vanadyl complexes, and 10 μg/mL actinomycin D) and incubated in the dark at 28ºC for 30 min with a gentle shaking at 50 rpm.
4. Nodule slices in the enzyme solution were passed through three-layer cheesecloth to filter out the small tissue debris and bacteroids. The residues retained on the cheesecloth were collected and washed three times by the cellulase and pectinase-free enzyme solution.
5. Then the nodule slices were further incubated in a 15 mL enzyme solution in the dark at 28ºC for 2.5 h without agitation, only with occasionally shaking in the last hour to accelerate the release of nodule protoplasts.
6. The solution containing released nodule protoplasts was then filtered with a 100-μm nylon mesh filter into a 50 mL round-bottom centrifuge tube. The tube should be tilted during the filtration process to ensure that the filtrate flows along the wall to the bottom.
7. The filtrate from the previous step was filtered again with a 20-μm nylon mesh filter. Enriched nodule cells were retained on the filter, including both infected and uninfected cells (Figure S1C). These nodule cells were then suspended in solution A (20 mM 3-N-morpholino propanesulfonic acid (MOPS)-KOH (pH 7.5), 0.6 M mannitol and 5 mM $CaCl_2$, 10 mM ribonucleoside vanadyl complexes and 10 μg/mL actinomycin D). The nodule cell suspension was incubated on the ice to maintain cell viability and await further isolation.

Part B: Separation of infected and uninfected cells

8. The glass capillary tube (shown in Figure S1D) was pulled to make a filament tip using a micropipette puller (NARISHIGE, PC-100).
9. The infected cells were larger and with reddish-brown color. The uninfected cells were smaller and almost transparent. These two types of cells with distinct morphology were isolated under an inverted microscope (Olympus, CKX31) using the glass capillary tube.
10. The captured cells were released from the glass capillary tube to the PBS-BSA solution droplets using a mouth pipette shown in Figure S1E. Each group of cells was washed twice with the same buffer, then transferred to the cell lysis buffer provided by the company (Lifeint, Xiamen, China; http://www.lifeint.cn/).
11. After being briefly centrifuged at 4°C, the cell samples were pipetted 10 times in order to disrupt the cells.

12. The final lysate from around 50-100 cells for each sample was then immediately frozen in liquid nitrogen and stored at -80°C until further use for SMART-seq2 library construction.

Notes:

1. A clean environment is required to prevent the contamination or degradation of nucleic acids.
2. Nuclease-free pipettes and tips should be used throughout the whole process.
3. Mannitol needs to be sterilized by filtration.
4. The final lysate of nodule cells should be immediately frozen in liquid nitrogen.

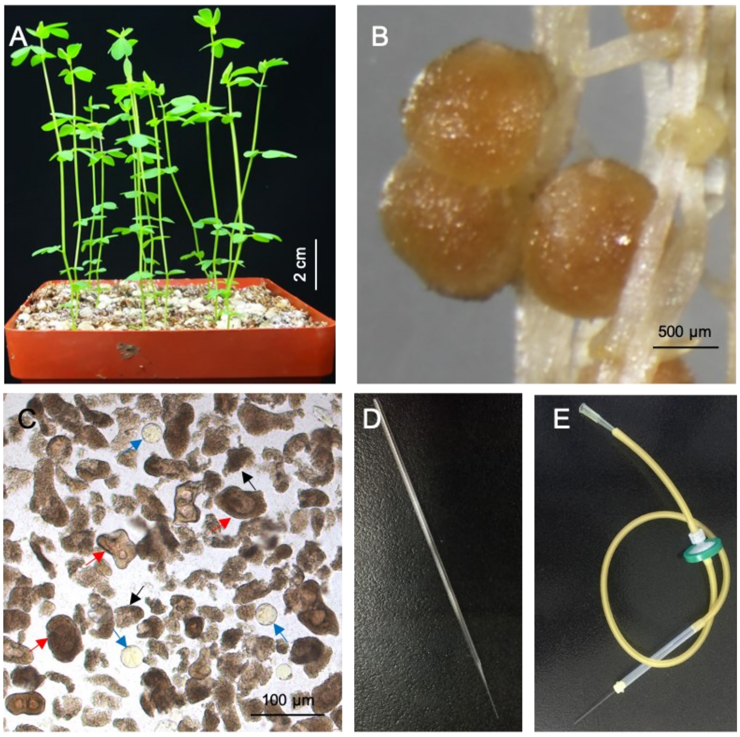

Figure S1. Representative materials and special tools used in this experiment.
(A) Plants of *Lotus japonicus* MG-20 at 4 weeks post-inoculation (wpi) with *Mesorhizobium loti* MAFF 303099. (B) Four-week-old nodules used for the preparation of nodule protoplasts. (C) Filtration-enriched nodule cells after enzyme digestion, containing infected cells and uninfected cells. Red, black and blue arrows indicate intact infected cells, broken infected cells and intact uninfected cells, respectively. (D) The glass capillary tube with a filament tip, which was used to entrap the nodule cells. The outer diameter of the tube is ~1 mm and the inner diameter of the tube is ~0.75 mm. (E) The mouth pipette used for the separation of single cell coupled with a glass capillary tube. Scale bars: (A) 2 cm; (B) 500 μm; (C) 100 μm.
